# Encephalomyelitis in a patient with monkeypox: an unusual complication

**DOI:** 10.1007/s13365-023-01121-7

**Published:** 2023-03-03

**Authors:** Daniel S. Marín-Medina, Laura Castilla-Gómez, Marcela Poveda, Leonora Ortiz, Lina M. Ariza-Serrano, Antonio Schlesinger-Piedrahita, Javier Torres-Zafra, Manuel Tapias-Agamez, Juan Pablo Osorio-Lombana, Gerson Arias-León, Edwin Silva

**Affiliations:** 1 Research Group NeuroUnal, Neurology Unit, Universidad Nacional de Colombia, Bogotá, Colombia; 2 Intensive Care Unit, Fundación Clínica Shaio, Bogotá, Colombia; 3 Department of Neurology, Fundación Clínica Shaio, Bogotá, Colombia; 4 Department of Infectious Diseases, Fundación Clínica Shaio, Bogotá, Colombia

**Keywords:** Monkeypox, Myelitis, Encephalitis, Encephalomyelitis, Acute disseminated encephalomyelitis

## Abstract

A new outbreak of monkeypox has been reported worldwide with CNS complications like encephalitis or myelitis being extremely rare. We present a case of a 30-year-old man with PCR-confirmed diagnosis of monkeypox who developed rapid neurological deterioration with extensive inflammatory involvement of the brain and spinal cord on MRI. Because of the clinical and radiological resemblance to acute disseminated encephalomyelitis (ADEM), it was decided to indicate treatment with high-dose corticosteroids for 5 days (without concomitant antiviral management due to lack of availability in our country). Given the poor clinical and radiological response, 5 days of immunoglobulin G were administered. During follow-up the patient’s clinical condition improved, physiotherapy was started and all associated medical complications were controlled. To our knowledge, this is the first reported monkeypox case with severe CNS complications treated with steroids and immunoglobulin in the absence of specific antiviral treatment.

## Introduction

Monkeypox is a zoonotic orthopoxvirus related to the smallpox virus. Originally, it was identified in humans in 1970 in the Democratic Republic of Congo, with sporadic outbreaks in the USA in 2003 and the UK in 2018 (Bunge et al. 2022). There is a new epidemic ongoing since the spring of 2022 occurring in many non-endemic countries, and because of the rapid growth of the epidemic, the Director-General of the World Health Organization declared monkeypox a Public Health Emergency of International Concern (PHEIC) on July 23, 2022 (WHO 2022). In the last months, most of the new cases of monkeypox have been reported in the Americas, but after vaccination, behavioral changes, and other public health efforts, it seems to be controlled in Europe and the USA. However, this trend has not been seen in South America and Africa, where cases continue to rise, and most of them are not recognized or tested properly (Reardon 2022). By the end of August, the moment of this case report, nearly 600 cases had been reported in Colombia and more than 7500 in Latin America (Mathieu et al. 2022). Although current data suggest a decline or stabilization in the rate of monkeypox infection, there is still uncertainty in many aspects of monkeypox biology to fully predict its future behavior and endemic potential (Reardon 2022). However, it is unlikely that the outbreak of monkeypox will lead to something similar to COVID-19.

Compared to smallpox, the clinical manifestations of monkeypox are usually milder and include fever, rash, flu-like symptoms, and lymphadenopathy. Most patients recover after 3 weeks, but a minority develop complications after 6 to 10 days of symptoms (Thornhill et al. 2022). CNS findings suggestive of inflammation are considered rare, with a low incidence of encephalitis and a mortality rate ranging from 1 to 10% depending on the clade involved (Shafaati and Zandi 2022). During the current outbreak, few fatal cases and very few cases of neurological complications such as encephalitis have been reported (Shafaati and Zandi 2022; Badenoch et al. 2022; Pastula et al. 2022).

The patient described in this report was diagnosed with Monkeypox and developed encephalitis and longitudinally extensive myelitis with severe motor, sensory, and cranial nerve function involvement during the second week of symptoms. The therapeutic course was based on clinical and radiological similarities with ADEM (acute demyelinating encephalomyelitis).

## Case description

A 30-year-old male, with a past medical history of unspecified lymphoproliferative disorder in childhood that was not active at the moment, presented with 7 days of fever, cough, fatigue, and a vesiculopapular rash in his lips and genitals. He reported having unprotected anal intercourse prior to the onset of symptoms. He was initially managed at home, but his symptoms worsened with weakness, difficulty swallowing, and inability to walk, which made him consult the emergency department. Monkeypox virus DNA was detected by real-time PCR of cutaneous lesion swabs, oropharyngeal swab, and serum. On day 1 after admission, he presented slurred speech and urinary retention, and was unable to move his legs. His clinical examination showed somnolence, bilateral miosis, bilateral peripheral facial weakness, dysarthria, paraplegia, positive bilateral Hoffmann sign, bilateral neutral plantar reflex, and a T6 sensory level for dull, pinprick, temperature, vibration, and proprioceptive sensations. Meningeal signs (including neck stiffness, jolt accentuation, and Brudzinski) were present.

**Fig. 1:**
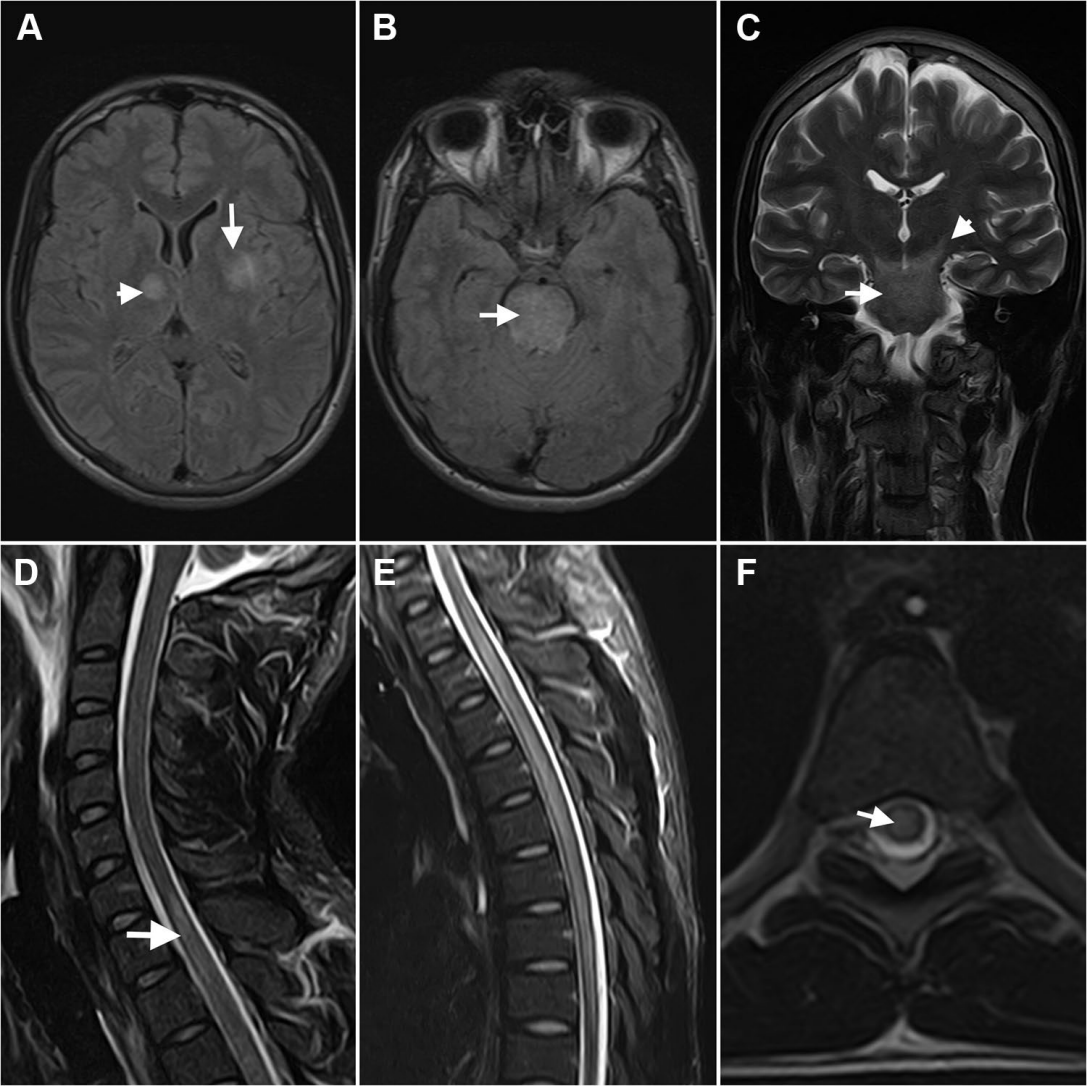
Brain MRI. **A** Axial FLAIR showing hyperintensities in the right internal capsule (arrowhead) and left basal ganglia (arrow); **B** axial FLAIR showing diffuse hyperintensity of the pons (arrow); **C** coronal T2 with extensive hyperintensities of internal capsules (arrowhead), midbrain, and pons (arrow). Spinal cord MRI. **D** Sagittal STIR showing hyperintensity below C7 level of the spinal cord; **E** longitudinally extensive hyperintensity along the entire thoracic spinal cord; **F** axial T2 with transverse hyperintensity of the spinal cord (arrow)

Laboratory tests on admission showed elevated white blood cells count, elevated C-reactive protein, with normal renal function and liver chemistries. HIV serology was negative. Contrast-enhanced brain MRI showed extensive hyperintensities on T2/FLAIR, predominantly in the white matter of brain hemispheres, and in basal ganglia, anterior thalamus, internal capsules, frontal medial cortex, cerebral peduncles, and the entire pons, some of which had restricted diffusion, but only a mild contrast enhancement, and no compression. Contrast-enhanced spinal cord MRI showed a T2/STIR hyper-intense longitudinally extensive transverse myelitis comprising levels T1 to T12, with no enhancement or compression (Fig. 1). The lumbar puncture showed normal opening pressure (20 cm H_2_O), a clear CSF with mild elevated protein and lymphocytic pleocytosis (Table 1). Monkeypox virus real-time PCR in CSF was negative. Transverse myelitis laboratory workup was performed, with serum anti-AQP4 and anti-MOG negative for demyelinating etiologies, B12 vitamin, and other nutritional deficiencies were excluded, and infectious etiologies were also excluded through CSF analysis (Table 1).

**Table 1:** Results of cerebrospinal fluid (CSF)

Colorless	Leukocytes: 57 cells/mm^3^	ZN: negative
CSF glucose: 43 mg/dL	Mononuclear cells: 80%	TB-PCR: negative
Blood glucose: 75 mg/dL	Erythrocytes: 0 cells/mm^3^	India ink: negative
Glucose ratio: 0.57	Gram staining: negative	VDRL: negative
Proteins: 95 mg/dL	Bacterial culture: negative	TB culture: negative

Film array: *Escherichia coli* K1, *Haemophilus Influenzae*, *Listeria monocytogenes*, *Neisseria meningitidis*, *Streptococcus agalactiae*, *Streptococcus pneumoniae*, *Cytomegalovirus*, enterovirus, HSV-1, HSV-2, HHSV-6, parechovirus, varicela zoster, and *Cryptococcus neoformans/Gatti*: not detected

*ZN* Ziehl-Nielsen, *TB-PCR* Mycobacterium tuberculosis polymerase chain reaction, *HSV* herpes simplex virus 1 and 2, *HHSV-6* human herpesvirus 6

ADEM was suspected due to clinical and radiological findings. Initially, he received symptomatic and supportive measures, but subsequently, he developed progressive loss of consciousness and respiratory failure, requiring invasive ventilatory support. Unfortunately, there were no antivirals for monkeypox available in Colombia by the time the patient was admitted; therefore, he was treated with systemic corticosteroids (methylprednisolone 1 g QD) for 5 days without concomitant antiviral treatment.

Patient’s neurological symptoms progressed in the following days with an abnormal conjugate gaze to the left with inability to abduct the right eye and nystagmus in the left eye, the bilateral peripheral facial palsy persisted, and he still did not have any respiratory drive. A new MRI showed increased hyperintensity of the previously involved structures such as the pons, midbrain, internal capsules, and basal ganglia, with a subtle increase in the size of the lesions.

It was decided to start adjunctive treatment with intravenous immunoglobulin (0.2 g/kg QD). On day 3 after initiation of IVIG, the patient presented with urinary tract infection that was treated with meropenem with an appropriate clinical response. The patient was weaned from mechanical ventilation and successfully extubated. After 4 weeks of the patient’s arrival and 3 weeks following completion of treatment, the clinical condition of the patient improved, with complete resolution of consciousness, he was able to communicate, cranial nerve involvement showed improvement in ophthalmoparesis, facial weakness and speech, sensory level was in T10, and all associated medical complications were controlled.

## Discussion

To the best of our knowledge, at the time of this report, this is the third case of acute dysseminated enchephalomyelitis in patients with monkeypox (Pastula et al. 2022), and the first managed without concomitant antiviral therapy. The neuroinvasive ability of Monkeypox virus in humans and a few cases of encephalitis have been reported in previous case series: one 3-year-old girl with fatal outcome in the Congo Basin, three patients in Nigeria, and a 6-year-old girl in the United States outbreak in 2003 with complete recovery after 1 month (Pastula and Tyler 2022). Normally, encephalitis is an unusual complication of monkeypox virus infection; in fact, a non-peer-reviewed systematic review by Badenoch et al. estimated a prevalence of 2% [0.5–8.2%] (Badenoch et al. 2022).

Encephalomyelitis may be an expected complication of orthopoxvirus infections. Postvaccinal encephalomyelitis (PVEM) is an uncommon but classically reported complication in patients receiving the smallpox vaccine. As in the patient in this report, neurological complications typically occur in the second week of symptoms. Patients present with evidence of encephalitis, myelitis, and meningitis. The cerebrospinal fluid (CSF) of patients with PVEM may exhibit an elevated protein concentration and lymphocytic pleocytosis, but it could be normal in up to 50% of patients. Investigation of this condition has rarely demonstrated direct viral involvement, and the most accepted hypothesis is a molecular mimicry phenomenon between viral proteins and myelin (Abrahams and Kaufman 2004).

Direct CNS infection was not demonstrated because real-time PCR of monkeypox virus in CSF was negative. Additionally, other CNS viral infections and immunological conditions, including paraneoplastic and antibody-mediated encephalitis, were ruled out. After the exclusion of this differential diagnosis, immune-mediated demyelination was considered due to clinical and radiological findings. ADEM is a form of acquired demyelination described in patients, most of them children, who had previously suffered from different kinds of infections, including smallpox. CSF shows mild lymphocytic pleocytosis and elevated protein. Serum MOG (myelin oligodendrocyte glycoprotein) antibodies could be negative in almost 40% of the cases (Otallah 2021).

After a risk–benefit assessment, it was decided to start a therapeutic course with corticosteroids following the ADEM treatment guidelines (Alexander and Murthy 2011). Considering the potential of a virological rebound with the use of corticosteroids, adjunctive treatment with cidofovir was considered. However, it was not possible because of the lack of availability in Colombia of any specific antiviral for monkeypox. IVIG treatment according to ADEM guidelines was subsequently started because of the clinical worsening. The clinical improvement seen in this patient after the use of IVIG, without major safety concerns, may suggest this therapeutic intervention for future cases; however, experimental evidence is required.

There were several challenges in the medical care of this patient: the rapid neurological deterioration and severe central nervous system involvement, the scarce information on the management of this type of complications, and finally, the absence of specific antiviral drugs in Colombia. There is no specific treatment for Monkeypox virus infection in humans; however, there are some pharmacological interventions to treat severe cases, approved for compassionate use, namely tecovirimat, brincidofovir, cidofovir, and vaccinia intravenous immunoglobulin. With new cases of monkeypox being reported every day around the world, it is likely that more severe cases with neurological complications arise.

Future research prospects in the aspects of monkeypox infection span from the basic mechanism of transmission, possible reservoirs, response to vaccination, and potential of mutagenicity, to clinical and public health aspects such as the implementation of screening tools, identification and early isolation of suspected and confirmed cases, and changes in behaviors and social stigmatization of populations at high risk for exposure (Reardon 2022). With this information, we hope that the scope of the infection could be delineated, and interventions could be addressed on a rationale and broader level.
